# Learning sites for health system governance in Kenya and South Africa: reflecting on our experience

**DOI:** 10.1186/s12961-020-00552-6

**Published:** 2020-05-11

**Authors:** The RESYST/DIAHLS learning site team, E. Barasa, M. Boga, N. Kagwanja, S. Kinyanjui, C. Molyneux, M. Nyikuri, J. Nzinga, B. Tsofa, D. Waithaka, E. Waweru, O. Abdulahi, T. Malingi, A. Omar, B. Mazoya, H. Leli, C. Mataza, L. Brady, S. Cleary, J. Daire, L. Gilson, U. Lehmann, N. Schaay, H. Schneider, V. Scott, S. Ellokor, P. Olckers, A. du Toit, S. Choonara, J. Goudge, N. Nxumalo, N. Madzudzo, S. Hlahane

**Affiliations:** University of Cape Town, Cape Town, South Africa

**Keywords:** Co-production, Embedded HPSR, Action learning, Health system governance, Decision-making, Micro-practices of governance

## Abstract

**Background:**

Health system governance is widely recognised as critical to well-performing health systems in low- and middle-income countries. However, in 2008, the Alliance for Health Policy and Systems Research identified governance as a neglected health systems research issue. Given the demands of such research, the Alliance recommended applying qualitative approaches and institutional analysis as well as implementing cross-country research programmes in engagement with policy-makers and managers. This Commentary reports on a 7-year programme of work that addressed these recommendations by establishing, in partnership with health managers, three district-level learning sites that supported real-time learning about the micro-practices of governance – that is, managers’ and health workers’ everyday practices of decision-making.

**Paper focus:**

The paper’s specific focus is methodological and it seeks to prompt wider discussion about the long-term and engaged nature of learning-site work for governance research. It was developed through processes of systematic reflection within and across the learning sites. In the paper, we describe the learning sites and our research approach, and highlight the set of wider activities that spun out of the research partnership, which both supported the research and enabled it to reach wider audiences. We also separately present the views of managers and researchers about the value of this work and reflect carefully on four critiques of the overall approach, drawing on wider co-production literature.

**Conclusions:**

Ultimately, the key lessons we draw from these experiences are that learning sites offer particular opportunities not only to understand the everyday realities of health system governance but also to support emergent system change led by health managers; the wider impacts of this type of research are enabled by working up the system as well as by infusing research findings into teaching and other activities, and this requires supportive organisational environments, some long-term research funding, recognising the professional and personal risks involved, and sustaining activities over time by paying attention to relationships; and working in multiple settings deepens learning for both researchers and managers. We hope the paper stimulates further reflection about research on health system governance and about co-production as a research approach.

## Background

Since the early 2000s, there has been growing recognition of the importance of governance to health system performance in low- and middle-income countries (LMICs). Early work provided important conceptual lenses for how to think about governance [1] as well as assessment frameworks [2]. Subsequent empirical work emphasised the critical role of governance in sustaining well-performing health systems [3], and a more recent analysis piece concludes that “*Governance is central to improving health sector performance and achieving Universal Health Coverage*” ([4], p. 1).

However, it is less clear how to strengthen health system governance and researching governance issues is far from straightforward. Indeed, in 2008, the Alliance for Health Policy and Systems Research (AHPSR) argued that ‘governance and accountability’ was a neglected research issue as a result of conceptual and design challenges as well as the political sensitivity of such research [5]. The AHPSR made three methodological recommendations for governance research, namely (1) use qualitative approaches, such as participatory approaches, participant observation, historical cases and processes, and institutional analysis; (2) implement multi-country field research programmes to share analytic approaches, address shared questions and share experience of addressing the practical challenges of such research; and (3) undertake such research with engagement from, and addressing specific questions identified with, policy-makers and managers.

Following on from the third recommendation, the AHPSR also argued that “*work on governance and accountability has the potential to gather the actors that contribute to health systems to not only generate new knowledge, but also to generate the dialogue, self-reflection and analysis that more directly uses this knowledge for policy change and offsets policy opposition*” ([5], p. 10). It would later call this type of work ‘embedded research’ [6], which in higher-income country discussions is also called ‘co-production research’ [7]. As others have also noted, because governance strategies are context specific, as they are “*rolled out, they should be linked to careful research that both enables learning as to what works, and facilitates fine-tuning and adaption of the strategy*” ([4], p. 4).

Against this background, the Resilient and Responsive Health System (RESYST) consortium proposed and implemented a 7-year programme of research (2012–18 https://resyst.lshtm.ac.uk/) that included a particular focus on governance and leadership. Reflecting the AHPSR recommendations above, this programme of qualitative governance research was implemented through iterative cycles of research and reflection undertaken collaboratively with health managers in three ‘learning sites’. One was located in Kenya and supported by researchers at the Kenyan Medical Research Institute–Wellcome Trust Programme (KWTRP) and two were located in South Africa, one supported by researchers at the Centre for Health Policy, University of Witwatersrand, and the other by a collaborative team from the School of Public Health and Family Medicine, University of Cape Town, and the School of Public Health, University of the Western Cape.

Our work was founded on the understanding that governance is a “*dynamic and complex process, rather than a normative health system goal achieved through the architecture and design of accountability and regulatory frameworks*” ([8], p. 1–2), [9]. Our research and intervention work, as detailed in Box 1, specifically investigated the micro-practices of governance, that is, managers’ and health workers’ everyday practices of decision-making. In addition, as discussed further below, we have engaged in policy and management discussions at higher levels of the health system in both countries, supported cross-country engagement and learning, and reached a wider audience through using the research in teaching programmes, publications and presentations at international conferences.

Box 1 The learning site research programme: understanding and supporting district-level micro-practices of governance Across sites, our research programme has had two main areas of focus.

First, we have extended global thinking by generating insights into the dynamic nature of district health systems. We have investigated (1) the experiences and views of those working within these systems [10, 11]; (2) the importance and challenges of mid-level leadership as well as community engagement [15]; (3) the routine practices of hospital priority-setting in Kenya [16] and, in South Africa, of human resource performance management [19] and information use for decision-making [20]. In Kenya, we have specifically investigated, in real time, the process and impacts of devolution in Kenya [21, 22] and the 2017 health workers strike [25]. Cross-site learning has, meanwhile, examined the shared experiences and realities of accountability processes within district health systems [26] and, unusually, generated insights into the nature of chronic shocks experienced at this level, how health systems respond to those shocks as well as the nature of everyday health system resilience [27, 28].

Second, across sites, we have also supported and tracked ‘home grown’ interventions to strengthen district health systems. So far, we have reported on South African interventions to sustain collaborative working arrangements [29] and to strengthen leadership at this level [30, 31]. We are currently writing up a newer wave of work across sites, which has both taken forward leadership support activities (Nzinga J, Boga M, Kagwanja N, Waithaka D, Barasa E, Tsofa B, Gilson L, Molyneux S: Strengthening health system governance: the potential of innovative leadership development in supporting health managers’ leadership capacities, unpublished) and, testing insights derived from earlier work, sought to deepen understanding of everyday resilience and the role of leadership in nurturing it [32].

### Learning site activities

#### Where and with whom did we work?

The learning sites are all located within health districts, as nationally defined, with work conducted at district and sub-district levels across sites as well as in primary health care (PHC) facilities and, in Kenya, district hospitals. These sites are Kilifi County, Kenya (formerly Kilifi District); Mitchell’s Plain, Cape Town, Western Cape province, South Africa (with work in the wider Area South, Cape Town, in 2017–2018); and Sedibeng District, Gauteng province, South Africa. There are differences in the geographic and socioeconomic context of each site [28] (and online supplementary material, 10.1136/bmjgh-2016-000224). Kilifi is a relatively poor rural area in Kenya, whereas the South Africa sites are located in urban areas and are relatively well-off compared to national averages (although Mitchell’s Plain sub-district is relatively poor within the wider City of Cape Town). From a health system perspective, meanwhile, PHC utilisation levels were similar across sites and PHC performance, as judged by antenatal care utilisation levels, was reasonable but showed room for improvement everywhere.

These areas were selected as learning sites based on the prior history of engagement between the research teams and local health managers as well as on their geographic proximity to the researchers’ organisational locations (Box 2). By providing a platform of trusting relationships and enabling the frequent presence of researchers in the sites, these features allowed the learning site work to be initiated and sustained over time. Critically, they enabled the research teams to implement their activities in a flexible manner, responding to opportunities and challenges that arose over time as well as managerial views, supporting, in turn, the deepening of our relationships. The research teams were multi-disciplinary, comprising people with different social science backgrounds and varying depths of health policy and systems research (HPSR) experience as well as health professionals. Although, across sites, there was some turnover over time in both the research and managerial teams, the continuity of key researchers and managers within a sustained platform of relationships provided the base from which to draw in new team members.

Box 2 The history of the learning sites *Kilifi County, Kenya*

In the Kilifi learning site, the relationship between the Kenyan Medical Research Institute – Wellcome Trust Research Programme (KWTRP) health policy and systems researchers and health managers has grown and evolved overtime. The KWTRP as a whole is a large health research programme that was established over 30 years ago and deliberately embedded within the Kilifi health system. Although initially focusing on biomedical research, health policy and systems research (HPSR) work was initiated in the early 2000s in collaboration with local health managers. Towards the end of that decade, a former district health manager (BT) took up a training and research role within KWTRP, helping to strengthen the overall collaboration and overcome communication barriers. Coinciding with the initiation of Resilient and Responsive Health Systems (RESYST)-funded research, his appointment also provided an opportunity for more structured collaboration between the KWTRP HPSR team and Kilifi health managers in a programme of research aimed at learning about, and strengthening, the performance of the local health system.

*Mitchells Plain, City of Cape Town and Metro DHS, Western Cape Province, South Africa*

Research activities in this learning site began in 2010, before RESYST initiation, supported by the District Innovation and Action Learning for Health System Development (DIALHS) collaboration funded by the Atlantic Philanthropies. The DIALHS collaboration comprised two university HPSR groups (in the Universities of Cape Town and the Western Cape) and managers from the provincial and local government authorities, both responsible for primary healthcare provision in the City of Cape Town. Mitchell’s Plain is a lower income area of Cape Town, within half an hour’s drive of the two universities. In 2017/2018, work was extended to Area South, a larger area within Cape Town. The Universities’ track record of district health system research and health management training, as well as personal relationships between researchers and health managers, enabled the establishment of the learning site. The DIALHS focus on health system governance was also seen as timely as the provincial government’s district health system was being established at the time of project development and there was shared interest in strengthening district health management in Cape Town.

*Siyaqinisa (‘we strengthen together’) learning site, Sedibeng, Gauteng Province, South Africa*

Sedibeng District is located about an hour’s drive from Johannesburg, where the Centre for Health Policy, University of Witwatersrand, is located. RESYST-supported learning site research in Sedibeng was initiated in 2014. However, the collaborative relationship between the Centre for Health Policy and the district dates back to 2008, before RESYST, when initial activities were focused on strengthening the District Health System through Comprehensive Primary Health Care. Building on the platform of the earlier activities and relationships, the learning site work was developed through an initial process of engagement and with a particular focus on strengthening leadership and management within the district. Some detailed work was specifically conducted in Emfuleni sub-district, where the core team of senior managers are located. The district manager was central to brokering interactions with the research team over time and, for learning site work, in providing access to other district managers and staff.

#### What did we do?

Table 1 provides details about how our core activities were implemented collaboratively over time with local health managers in each site. There were some differences between sites in our initial approach to the research and in the balance between more traditional, protocol-driven research activities and a more flexible, action learning process. Nonetheless, our activities [33] were commonly founded on principles of action learning and co-production drawn from outside the health sector and outside health policy and systems research [34–37].

**Table 1:** Core learning site activities

	Kilifi	Mitchell’s Plain	Sedibeng
Initial research proposal (focus and activities)	Proposed a traditional but quite broad research project addressing the functioning of planning, management and accountability processes in the district (county). The science/ethics proposal included data collection through observation, document review and interviews, also allowing for reflection on research findings and identification of additional specific case studies with managers; therefore, the proposal enabled the research team to have increasing flexibility in responding to emerging, manager-informed questions	Proposed an action learning process involving researchers working with local health managers and staff to (1) consider local needs and opportunities; (2) identify key entry points through which to strengthen the district health system (considering planning, leadership, management and monitoring processes, concern for community engagement, inter-sectoral engagement); repeated cycles of planning, implementation/ practice, reflection and evaluation, learning, and revision were outlined in the science/ethics protocol, with data collection through observation, document review, interviews and reflective practice discussions	Proposed an action learning process involving district health managers and researchers; included traditional research activities to learn about district planning, management and accountability processes (including, specifically, staff performance management) and daily challenges involving interviews, document review and observations; the science/ethics protocol also allowed for feedback to district managers and collective reflection to generate new rounds of activities
Subsequent activities	As our learning evolved, a second science/ethics protocol focused more broadly on the micro-practices of governance at sub-national level (county, sub-county and facility level), considering leadership practices, organisational relationships and their underpinning values; additional research foci included programme-based budgeting, facility financing and the participatory development of a leadership intervention Cycles of reflection and planning with managers remained the core research approach.	Annual cycles of action, learning and reflection continued over time in line with the original protocol Specific sub-studies were also developed with their own research protocols, where they entailed engagement with a new set of respondents or a new data collection approach (e.g. inquiry into primary healthcare facility managers’ experiences involving journaling [10]) Over time, the annual cycles of action and learning also included support for managerially led interventions focused on management processes and leadership development interventions	Based on earlier learning, a second science/ethics protocol focused on initiating and conducting monthly leadership support intervention workshops with senior and middle level managers, with the support of an external facilitator; this was accompanied by traditional research activities such as diaries and observations to understand and track experience over time
	The most recent phase of work across sites was guided by a shared umbrella proposal, supporting us to test our emerging insights into everyday resilience [28] across sites using a common conceptual framework, with activities adapted to each site as appropriate		
Cycles of reflection among researchers	Reflection meetings were held every 1–4 months, with more regular discussions in busy data collection periods; in meetings, de-brief on learning across a series of sub-areas of interest; share information on cross-cutting contextual issues and observations; share theoretical and empirical work to assist analysis; discuss strategies for engagement with managers, reflecting on our varied expertise and experience; specific sessions were dedicated to ethical dilemmas in response to experience	Regular reflection meetings were held monthly in the initial years and, subsequently, around every quarter or more frequently as activities required; these meetings included de-briefing on observations and engagements in the learning site; discussion of ethical dilemmas; review of relevant theoretical and conceptual work to assist analysis; identifying and reviewing emerging insights from analysis; planning further activities	Regular reflection meetings were conducted monthly, including to de-brief on engagement and emerging developments in the learning site and to generate potential strategies to manage emergent developments (such as ethical dilemmas); during the second phase, meetings were held prior to and after each leadership intervention workshop throughout the intervention cycle
Cycles of reflection with managers	Once/twice a year, formal reflection meetings were held to allow feedback of emerging findings and their discussion as well as to identify key priorities and interests moving forwards; regular informal, continuous interactions between researchers and core managers, to track contextual issues and co-produce research outputs	Annual reflection meetings held to feed back on work conducted and insights generated as well as to identify next steps of research and other new activities Regular informal and continuous discussions with core managers to track developments and activities within the site as well as around co-production of research outputs	Initially, regular informal discussions with district manager to brief and clarify emerging findings and developments, and to identify key areas to address in new activities; subsequently, de-briefing discussions with district manager at key points of the leadership intervention to reflect on developments and the ongoing activities within the site
External sounding boards and concern for ethics	Annual reporting process to check science and ethics across sites involving external actors; in addition, responding to emerging concerns, two ethics reflection sessions were organised at annual RESYST meetings to support one another in ensuring ethics in practice in each site (see also [39])		

The table highlights six common features of our activities.

1. Multiple research and intervention activities were implemented over time in each site:
  The research comprised specific qualitative research projects as well as longitudinal processes of observation and inquiry. The interventions, several of which focused on leadership development [31], were either led by learning site managers or by external facilitators in agreement with these managers, rather than being externally driven.
2. Collective leadership by the research team and local managers for the iterative development of all activities:
  Each new activity was identified collectively through cycles of research, reflection and reporting, and was then supported by the team of researchers and health managers. Over time, previous rounds of research then provided the platform for newer research, supporting deeper inquiry into specific issues/experiences and allowing some external events (such as devolution in Kenya) to be tracked over time. In addition, home-grown interventions evolved out of the research and were tracked as implemented.
3. Reflective practice was the core feature of all learning site activities, supporting sense-making and learning:
  Researchers met together to reflect on observations and emerging insights, and reflective meetings between researchers and managers allowed emerging insights to be tested to deepen understanding. It was also common practice to deepen reflection by bringing theoretical and conceptual frameworks and questions as well as experience from the other sites into the conversations between researchers and managers.
4. Multiple data sets were generated in each site:
  Formal data collection processes around specific research questions were complemented by data generated through regular observation and participation in learning site life and from reflective discussions [33].
5. Co-production of knowledge and action was the hallmark of our work:
  This was implemented through collective leadership and reflective practice. It is also demonstrated in the co-authorship of formal papers, in the implementation of new managerial activities across sites and in the development of policy lessons.
6. Paying careful attention to our research approach and related ethics issues:
  Initially, we outlined and discussed the core features of our work in our institutional and national ethics review proposals. Subsequently, we regularly reflected on the overall approach [38] and the ethical issues and dilemmas we encountered as it evolved (Scott V, Xapile Z: Negotiating ethics at the frontline of HPSR practice: participatory action research with health managers, unpublished), [39]. We also established an additional informal annual science/ethics review group to provide us with an independent review of our evolving learning site ideas and activities, including the need to submit amendments or changes to the formal ethics’ review panels.

#### How did we work?

**Fig. 1:**
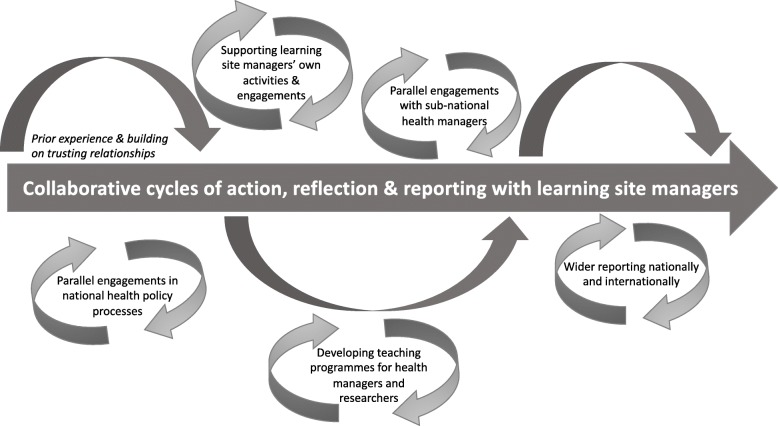
The package of learning site activities

Around the core activities, as shown in Fig. 1, an additional set of engagements within and outside the learning sites were important both in sustaining relationships and in sharing the learnings from the primary research and intervention work in each site more widely. These activities were developed in response to site-, organisation- and country-specific opportunities and so inevitably varied, as described in Table 2. However, they all sought to strengthen the researcher–manager relationships underpinning learning site activities, including by allowing better understanding of each other’s organisational worlds. For example, researchers’ engagement in managerial activities brought wider insights into the demands of health management. Meanwhile, managers’ involvement in conferences and teaching provided insights into wider academic debates and practice. The activities outlined in Table 2 also allowed the research learnings to be shared more widely at national and global levels. Wherever possible within national settings, efforts were made to use the learning site work to inform wider policy and practice discussions. As the table notes, in some instances, these efforts were linked to specific formal policy processes. In other instances, more informal engagements allowed researchers, as in Kenya, to offer strategic advice on various issues or, as in Mitchell’s Plain, informed broader conceptual thinking of relevance to policy discussions. In Sedibeng, engaging at higher system levels appeared to be more challenging than elsewhere, perhaps because instability within the district governance structures focussed the managers and researchers on supporting this district’s own development. However, across all sites, research findings were also used within formal teaching programmes, allowing learning site experience to be disseminated more widely to audiences of public sector managers as well as to future health policy and systems researchers. Finally, at the global level, the learning site approach and experiences were shared through collective RESYST engagement at the 2016 and 2018 Global Health Systems Research symposia, and through peer-reviewed journal papers.

**Table 2:** Activities to extend and deepen learnings

	Kilifi (Kenya)	Mitchell’s Plain (Western Cape province, South Africa)	Sedibeng (Gauteng province, South Africa)
Engaging at higher levels of the system	Research team members hold formal and informal policy advisory positions at county and national level, drawing the research findings into wider policy debates and discussions; for example, EB sits in different national committees that are developing guidelines and guiding the implementation of the national Universal Health Coverage agenda; BT sits in the national health sector planning Thematic Working Group and, in 2018/2019, was invited to support a multi-stakeholder team working on gathering evidence-based lessons for strengthening health sector devolution in Kenya; within the county, managers shared lessons upwards with more senior managers	Initially, regular meetings were held between researchers and senior provincial and local government managers to feed back insights from the learning site work and secure support for future activities Subsequently • Researchers were engaged by senior provincial (and, occasionally, national) managers around specific areas of learning relevant to parallel policy processes (e.g. leadership development, relational governance) • Learning site managers themselves shared lessons both upwards (with senior managers) and sideways (with colleagues) Wider researcher–policy-maker engagements in the province also drew on learning site insights and supported the development of shared understandings on governance issues [40]	Challenges experienced in engaging at higher levels of the system; despite the long-term relationship between the research team and district managers, opportunities to extend this interaction upwards in the health system did not materialise, perhaps due to the district’s internal challenges, which led the collaborative work to focus on supporting sub-district development
Responding to managerial requests for support, within and outside the learning site	Less formal requests included, e.g., requests for transport from managers given lack of funding for supervisory visits to facilities More formal requests included supporting/chairing the development of the county and regional health plans	Largely informal requests, e.g., to make presentations on health system governance issues to wider groups of managers, facilitate meetings discussing health systems issues and provide advice on specific problems and issues	Informal requests to facilitate budget planning meetings for individual coaching
Involvement of learning site managers in wider activities	Support for managers’ engagement in Africa-wide discussions on leadership development; study visits and short course HPSR training in South Africa and Kenya; nomination of a manager for global award (Heroines of Health)	Support to primary healthcare facility and sub-district managers to attend and report on work in national and international research conferences; support for managerial exchange visit to Kenya	Support for district manager’s participation in short-course health policy and systems research training and conference attendance
	Collective engagement with and presentation at 4th and 5th Global HSR Symposia (2016, 2018) (noting that it would be difficult for health managers to fund/attend without research team support)		
Linking research to teaching	Learning site research insights have been drawn into short-course training for managers within Kenya and into a global health masters course in Oxford University	Initial action learning proposal included the specific intention to draw research findings into health management teaching Direct use of research insights in short course health management training as well as postgraduate health management and systems training (e.g. University of Cape Town post graduate Diploma in Health Leadership and University of Cape Town/University of the Western Cape Masters in Public Health programmes)	Insights from the learning site work are feeding into Centre for Health Policy teaching on the University of the Witwatersrand Master’s in Public Health training programme
	Learning site experience has been used in teaching cases around complex health systems, within open access training curricula available from the Collaborative for Health Systems Analysis in Africa, CHEPSAA www.hpsa-africa.org		

### What are the achievements and challenges of the learning site approach?

#### Achievements

Across all three learning sites, researchers and health managers involved in this work agree that the approach has generated value for understanding health system governance and for strengthening it.

In annual reporting to the Department for International Development, United Kingdom – the RESYST funder – the learning sites reported policy impact at both instrumental and conceptual levels [41] across countries. As Box 3 summarises, there were several instances of policy development and practice change resulting from learning site work (Box 4), with the latter also observed by the research teams. Moreover, as already discussed (Table 2), the links to the research partners’ teaching activities as well as our collective publications and participation in global conferences have enabled us to share our experience more widely. These activities support a multiplier effect in terms of audience size; additionally, teaching, in particular, allows a deeper engagement with the learning site experiences.

As illuminated in Box 4, learning site managers have found particular value in the reflective practice principles and practices to which they have been introduced, and that have allowed them to stand back from the persistent challenges they face and think again about how to address them [42]. Across sites, the managers’ growing understanding of the health system, in particular, as comprising both software and hardware [12] has also brought them valuable insights into the system in which they work and how to lead change within it. They judge that these practices and ideas have supported them to adopt a more proactive and confident approach to management and has generated the positive energy required to tackle problems. They have valued the safe spaces that have been created through reflective practice, allowing difficult conversations and bringing together different actors in the system to work together [29]. They have appreciated the training and mentorship that they have received in leadership and communication practices, for example, developing new skills they now regard as essential for their management positions. They judge that these interventions were well tailored to their needs precisely because they emerged from prior rounds of learning site research and understanding (Nzinga J, Boga M, Kagwanja N, Waithaka D, Barasa E, Tsofa B, Gilson L, Molyneux S: Strengthening health system governance: the potential of innovative leadership development in supporting health managers’ leadership capacities, unpublished), [31]. In some instances, researchers have also been valued as an external resource on which managers can draw to offer advice about tackling problems (Table 2). Finally, the managers judge that the critical factor underpinning these valued outcomes has been the “*how of the learning site approach*”. The key features of the approach that they have identified are the efforts to address, and respond to, managerial needs and experiences; continued engagement over time and regular feedback focussed on “*how can we learn from this and not on this was done wrong*”; and a process in which managers were not just participants but also co-producers of knowledge and action, “*appreciated for the work we do*”. The development of strong relationships between managers and researchers was seen as central to the approach and its value, based on mutual respect and a growing understanding of each other’s contexts.

These relationships are also important to the researchers and the value they have derived from the learning site approach. For them, the value lies in:

- better understanding of the complexity of health systems and, in particular, emergence as an element of complexity [27, 43] as well as of the micro-practices of governance (Box 1). This was generated through observing change as it unfolds over many years, gaining insight into the experience and tacit knowledge managers use in daily decision-making and developing a more nuanced understanding of the contextual factors influencing this decision-making;
- the deeper insights generated when trusting relationships provide spaces for reflection and openness even on sensitive issues and experiences;
- being able to generate layers of understanding and knowledge; this was enabled through cycles of engagement over time involving observation, interviews, shared experiences and collective reflection and these, in turn, underpin the trustworthiness of the insights developed [33];
- opening up pathways to influencing practice and opportunities for wider policy influence through research uptake; this resulted from working hand-in-hand with managers and supporting them to take action to strengthen local governance processes as well as from sharing the experiences upwards and outward into formal policy processes, wider reporting and teaching activities;
- the opportunities provided through the platform of work to enable capacity strengthening – mutual learning – around health system governance and this form of embedded HPSR, for researchers at all levels of experience (including opportunities for master’s, doctoral and postdoctoral research);
- opportunities to generate new research questions and focal areas that are derived from past insights, so deepening and sharpening inquiry. For example, building on our understanding of everyday resilience [28], we have undertaken further work to test and develop our insights [32];
- opportunities to generate learning from comparisons across settings – given a common focus and approach in the learning sites. We have also demonstrated both the many common challenges district health systems face in different settings, and that there is much to learn across settings about how to respond to those challenges;
- opportunities to deepen and refine the overall learning site approach as an approach to embedded research – by sharing experience across the three sites, and among researchers and managers. We have, for example, learnt from each other about the importance of engaging managers at higher levels of the health system or about drawing learning site experience into teaching activities, to support wider impacts;
- deeper awareness of the ways in which the science and the ethics of this work is inter-twined, and of the need to ensure constant reflection on interactions and their implications in maintaining ethical practice. As researchers, we need to ensure transparency and respect in our engagements with our managerial colleagues, and to support longer-term learning in ways that minimise negative repercussions for individuals and organisations in terms of relationships and reputations.

Box 3 Examples of instrumental policy impact *Kilifi county, Kenya*

Early work by the learning site team identified the significant resource challenges faced by district hospitals and the central role of hospital user fees in assisting hospitals to cope with these challenges [16]. The hospital financing challenges were worsened by the loss of hospital autonomy over user fees resulting from the implementation of devolution and the new public finance management laws [21, 22]. These findings were fed back to county policy-makers and led to the enactment of a county level Kilifi Health Sector Facility Improvement Fund Act that has sought to return utilisation and management of user fees back to the hospitals. Since 2017, the learning site team has been involved in discussion and efforts to support the implementation of this Act.

*Western Cape province, South Africa*

The early phase of DIALHS learning site work generated insights into the leadership challenges and needs for district health system development at sub-district and facility level [10, 12, 13]. These insights fed into a wider process commissioned by the Western Cape provincial department of health, to support the development of its 2016 health leadership development policy and competency framework. The learning site team is now actively using these frameworks to shape its own leadership development programmes.

*Mitchell’s Plain health sub-district and Sedibeng health district, South Africa*

Managers have adopted new approaches to meeting management, including rotating the chair, managing time effectively, allowing small group discussion on specific issues, having opening and closing rounds to allow all voices to be heard – all of which they regard as opening up spaces for developing shared understanding and supporting service delivery.

Box 4 Managerial voices, learning site impacts

- “*we were looking at the health system as blocks, and the unlearning that took place … was really helpful*”
- “*we were able to step out the system … and that gave us a very different view*”
- “*we feel like we got a new lifeline to navigate the system*”*.*
- “*we have done management …*[but] *the old school management doesn*”*t work… here we learnt a new way*”
- “*in this programme you taught me that yes some things are not happening (in the department) but what is it that I can do to lessen the stress for myself and to lessen the stress for the people that I work with?*”
- “*I used to just complain about the new political players and how they are messing up our* [health management] *work but now I have learned that I can use my communication skills to actually talk with them and get some of the things that I need*”

Source: cross-site reflective discussions, Kilifi July 2018; Sedibeng learning site reflections 2018

#### Challenges

There are, of course, also challenges and limits to the learning site work. We highlight particular areas of critique here, linking our own experiences to a recent paper on co-production [7].

In an early evaluation of the overall RESYST programme of work, the learning site activities were criticised for implementing a programme of primarily descriptive and observational research that might neither produce generalisable lessons of “*verifiable policy relevance*” nor contribute to new knowledge. As Oliver et al. ([7], p. 5) note, a co-production researcher risks being seen as “*an academic lightweight, producing nothing of substance*”, “*being asked to answer questions which are dull, not novel (little contribution to the scientific literature), or not generalizable (focused on local issues)*”.

We recognise that we have not developed a traditional intervention ‘model’ for health system strengthening in other settings. Our modelling lies, rather, in our processes of learning, engagement, and intervention learning and, through them, we judge that we have made conceptual and instrumental [41] contributions to health system governance and strengthening health systems. The ‘thick description’ of the micro-practices of governance and everyday realities of health system managers and staff has added to the limited empirical knowledge base of health governance contexts and practices in LMICs (Box 1), with insights of relevance beyond the learning sites. It has also provided the platform for developing interventions rooted in those realities, rather than imposed on them from outside (be that from national level or by external actors). We have, then, developed leadership development interventions in these sites based on our initial research (Nzinga J, Boga M, Kagwanja N, Waithaka D, Barasa E, Tsofa B, Gilson L, Molyneux S: Strengthening health system governance: the potential of innovative leadership development in supporting health managers’ leadership capacities, unpublished), [31], that could be considered in other settings, and we have fed our research insights into our own and others’ leadership development activities. In addition, the co-production of these health system understandings itself supported learning site managers to adapt their own practices and to develop home-grown governance interventions. In complex adaptive systems such changes are seen as key steps in the process of adaptive learning important in nurturing emergent and sustained change [44, 45]. Finally, as previously noted, our research has evolved over time to allow initial insights about everyday resilience to be further tested and developed – a process of research we judge as simply necessary in investigating the contextually specific phenomena that represent the essence of governance in complex health systems. We look forward to publishing our revised learnings [32].

There are, nonetheless, professional and personal risks from this type of work [7]. It has not always been possible to work consistently and effectively with managers at higher levels of the health system to translate learnings into wider system gain. Tracking conceptual and instrumental impacts is not straightforward. The breadth of activities we have implemented (Fig. 1) goes well beyond formal research or teaching activities and not all are well valued by academic or research organisations. The activities have also been demanding because they have, purposefully, been implemented over a fairly lengthy period of time and connected to wider organisational activities, such as teaching, to support wider impacts. Finally, there has been personnel turnover in the research teams, and some managerial instability, particularly in Sedibeng, which has sometimes constrained our work. Although likely inevitable in any long-term research endeavour, managing relationships and research over time is part of the “*emotional labour of working collaboratively*” ([7], p. 5).

Yet, the longitudinal dimension of our work has allowed us to deepen our insights about the complexity of health systems and their governance, and how they change over time. Being embedded in a health system also assists in tracking how ideas and new ways of thinking feed into policy development or influence wider action, as managers use their positional power (see Boxes 3 and 4). Our fairly long-term funding has clearly been important to sustaining this work.

A further critique of coproduction research processes is that “*researchers risk being seen as partisan and/or lacking in credibility*” ([7], p. 5) where they are used to add legitimacy to pre-existing political positions or where they only report what is judged acceptable to policy/management partners. We recognise these issues as critical ethical issues and see them as a concern for all HPSR work, given its applied focus, rather than only for co-production research [46]. We have tried to pay active attention to managing the many sets of power relationships within our collaborations as part of our ethical and reflective practice. Perhaps we have been fortunate in rarely confronting situations of interpersonal conflict or disagreement between researchers and managers in any site. However, we have certainly discussed how and to whom to report some of our insights – less to avoid conflict, and more to encourage understanding and action. Yet, certainly, a risk of this form of research is in it being seen as biased and not independent – in part, itself a reflection of the enduring epistemological battles amongst health researchers [7, 47].

A final critique of our approach is that we did not purposefully evaluate whether and how we have had system impacts, and at what cost. We simply did not initially see our work as a system intervention. However, we do now recognise the potential value of addressing these criticisms in any future learning site work to allow deeper understanding of the overall approach. Careful attention would need to be paid to delineating and tracking the full set of relevant activities which, as summarised in Fig. 1, change over time; additionally, tracking the full range of possible impacts from these activities would have challenges. Simply resourcing an additional layer of such evaluation could also be difficult. Nonetheless, there would be value in testing and building the case for the potential of learning sites as an HPSR methodology for system impact.

## Conclusions

Whilst recognising the challenges identified, we suggest that our experience shows that this sort of long-term, embedded/co-production approach offers value for understanding health system governance, and addresses the AHPSR [5] recommendations for governance research. However, we do not suggest that our approach is the only way of doing such research.

Overall, we propose five key lessons from the experiences we have presented.

1. Through the learning sites we have been able better to understand the micro-practices of governance and the complex everyday realities of health systems in two countries. In line with wider work on bottom-up perspectives of policy change [48] and development thinking [44], we judge that these realities are vital to consider in developing system strengthening interventions that either work with the grain of existing institutional arrangements or, appropriately and knowingly, challenge those arrangements.
2. Through our work, we have seen the potential to change routine managerial practice by offering opportunities for sensemaking through a process of action learning and reflection. Managers lead change in their systems and welcome the opportunity to engage with ideas, such as the software of health systems, everyday resilience or complex health systems, that allow them to make sense of their world and how to act differently within it. Such emergent change is more widely seen as an essential element of transformative organisational change [27]; these insights also have particular value for how to think about, and do, leadership development in LMIC settings [31, 49].
3. Doing this sort of work, and seeking wider impact, requires entrepreneurial behaviour, supportive organisational environments and, at least some, long-term research funding. It cannot be seen purely as a researcher-driven endeavour and, for impact, must be linked to sustained engagements with higher level managers across the health system as well as to activities such as teaching, and various collective activities. It is not a project activity, but a longer-term programme of work, and there are professional and personal risks to it.
4. Initiating such activities is enabled by an existing platform of trusting relationships between researchers and managers, whilst sustaining them over time requires that continued attention is paid to maintaining those relationships. Relationships among researchers are also important. Trust and openness are critical. Being responsive to each other and being flexible, whilst also being able to set boundaries and navigate them, are necessary.
5. We found real value in working in multiple sites as the cross-site engagement deepened learning for managers and researchers – allowing comparison of different contexts and experiences, with recognition of the commonalities in experience. Whilst the micro-practices of governance are inevitably context specific, there are shared patterns and themes in these experiences across settings that provide the basis for new managerial practices and new ways of thinking about how to support them [25, 28].

We welcome further engagement with our experiences and reflections.

## Data Availability

Not applicable.

## Abbreviations

AHPSR: The Alliance for Health Policy and Systems Research
HPSR: Health policy and systems research
LMICs: Low- and middle-income countries
PHC: Primary healthcare
RESYST: Resilient and Responsive Health Systems
